# Psychiatry

**Published:** 1904-06-11

**Authors:** 


### PROGRESS IN MEDICINE AND SURGERY.

### PSYCHIATRY.

(*Continued from page* 175.)

**Acute Delirium.**—Although familiar clinically during a century of clinical observation and study, acute delirium has been little understood, and has been the subject of much indefiniteness and misunderstanding in psychological medicine. This is the more remarkable, says Dr. William B. Pritchard,[3] in that the clinical assemblage and course of symptoms at least seems to be both distinct and clean cut. While many authors of the English and American schools favour the term *acute* (or grave) *delirium* (Spitzka, Gray, Wood, Berkley), most French authors use the identical term delirè aiguè. Berkley states[4] in his text-book that acute delirium together with hallucinatory mental confusion (sub-acute delirium) are "forms of insanity which really do not belong to the idiopathic manias," though from superficial resemblance of symptoms both have in the past been classed as "mania." The difference lies in the fact that in subacute delirium (hallucinatory delirium) there is a departure from the character of mania in that a pronounced confusional delirium is noted in addition to the motor disturbances which themselves are produced in a state of automatic or dreamy sub-consciousness, and that in acute delirium the difference is still greater and is attended with pyrexia, profound physical and mental prostration and death within a period of a few days. The pathological condition of brain underlying acute delirium is obscure, but enough is known to state that an acute meningo-encephalitis, associated in many cases with a streptococcal, bacillary, or mixed infection is present. The disease is, however, one which is peculiar to persons of debilitated brain and body, occurring as it does in alcoholic, puerperal, post-febrile, and septicæmic conditions, or in persons who have been the subject of cerebral traumatism, including insolation, or in patients who have been the subjects of one or more attacks of insanity and are tending towards chronicity. The disease never occurs *de novo* in healthy subjects, and even among those of debilitated brain and body, as just referred to, it is rare. Krafft-Ebing could only collect 45 cases during his long experience as an alienist, Christensen met with it 33 times out of 1,800 asylum admissions, Bianchi and Piccino, during a period of several years, could report only 8 cases, and Jessen 5 cases, while Berkley found 3 cases only among 960 admissions. Dr. Pritchard records the following case:—A woman, aged 45, the mother of two children, of adipose and flabby physique and pale complexion, had been under treatment from time to time for a number of years for various ills, chiefly functional, nervous, and gouty disorders originating in, and aggravated by, a fretful domestic situation and dietetic indiscretions and excesses. There was a strong family history of insanity, a brother and an aunt dying of insanity. The patient was herself deficient in self-control and given to excesses, especially as regards stimulants, to her great worry and self-reproach, since she was otherwise an intelligent woman possessed of æsthetic tastes. Soon after the death of her husband, who was a man of wealth, she established relations with another man, and within a year gave birth to a child apparently healthy. This man eventually developed general paralysis, of which he died. He was undoubtedly a syphilitic subject but he did not seem to infect his wife, as neither she nor her offspring showed the slightest traces of direct or inherited syphilis. Five years of much distress and trouble, owing to the misdeeds of her husband and various legal and domestic worries made her health deteriorate. On December 10, after a stormy interview with a lawyer, she became much excited and had a severe "hysterical" attack. Within an hour she recovered, but on the following day she had a similar attack. "She responded to symptomatic treatment and for an hour discussed coherently and with perfect intelligence . . . though somewhat excited in manner. . . . The same evening she had another and similar attack. . . . Twenty-four hours later, having been meanwhile in normal condition, she suddenly developed a violent explosive maniacal delirium, shouting at the top of her voice, screaming, tearing her clothing into shreds, breaking the furniture . . . walking and talking, when not screaming, incessantly. . . . Her face was almost purple in its venous turgidity. . . . She was greatly excited, talking incessantly in a loud voice. Her language was grossly profane, obscene, and shameless beyond description. She talked of her persecutors, her innocent children, and of the vengeance she intended for her enemies. . . . One moment she prayed and the next she talked blasphemous filth. Much of the time she walked the floor with dramatic grandiose stride, her clothing in shreds and almost in the nude." After some time she grew calmer, and under treatment with hypodermic injection of hyoscine hydrobromate she became comparatively quiet, and was induced to take medicine and food. She was also given 10 grs. each of calomel and jalap, followed about an hour later by 50 grs. of bromide and 20 grs. of trional. The period of relative calm continued for nearly three hours, during which she slept for half an hour. Immediately on awakening she became violent and destructive as before. She bruised and injured her limbs and face against the walls of the room (which was bare of furniture), and was utterly beyond the control of two nurses. The following day, again under the influence of hyoscine, a period of calm was secured, and then bromide and trional were given, with the result that she slept for two hours. On awakening, her delirium began as before, and its intensity and violence were almost indescribable. From this day on to the fatal termination of the case on the sixteenth day her delirium was continuous, as were her violence and destructiveness, her obscene blasphemy of speech, indecency of conduct, obstinate insomnia and refusal of food. From the sixth to the twelfth day she slept less than two hours in the twenty-four, and had to be fed by the nasal tube. Occasionally she would drink a glass of milk with avidity, and then spit it into the face of the nurse. She expectorated freely during this period. She had visual hallucinations of her enemies, and of her husband, her children, and the Virgin. Scenes in court and interviews with lawyers were re-enacted. Her violence was so unabated that it became necessary to employ six nurses a day and physical restraint. The bowels were kept open with croton oil. On the tenth day incontinence of urine developed. The pulse was throughout 110 to 130, the daily temperature was from 102° to 103°, and a few days before death incontinence of fæces with diarrhœa developed. Vaso-motor disturbances were noted, but no trophic changes (bed-sores) followed. The pupillary and other reflexes were deeply altered and varied from time to time. At no time were there any convulsions or fibrillary twitchings. On the sixteenth day she suddenly collapsed, the pulse became thready and uncountable, the breathing was stertorous and laboured, and she passed into a state of coma and died in a very few hours. The necropsy of the brain removed four hours after death showed a normal dura-mater; the pial vessels were markedly injected, and the pia-arachnoid was markedly œdematous, the cortical capillaries were also engorged but no petechial hæmorrhages were observed. The fluid in the ventricles was clear, the choroid plexuses were congested and œdematous. To the naked eye and touch the cortex and white substance were firm and normal, the only change being the capillary congestion. Portions of the brains showed with Nissl's method the following microscopic changes: the larger pyramidal cells of the Rolandic area were ragged or eroded in outline, with marked destruction of the chromatic bodies; there was commencing vacuolation of the cell-protoplasm and eccentric displacement of the nuclei; and similar destructive changes were seen in the parietal convolutions and in the cerebellum where the Purkinje's cells were reduced to "mere shadows." The blood-vessels showed extreme distension but no infiltration or other appreciable change could be seen in the perivascular sheaths. No bacteriological examination was made.

[3] Jour. of Nerv. and Mental Diseases, March 1904. [4] Mental Diseases, p. 157.

*(To be continued.)*

